# Harmonized Collaborative Validation of Aflatoxins and Sterigmatocystin in White Rice and Sorghum by Liquid Chromatography Coupled to Tandem Mass Spectrometry

**DOI:** 10.3390/toxins8120371

**Published:** 2016-12-13

**Authors:** Hyun Ee Ok, Fei Tian, Eun Young Hong, Ockjin Paek, Sheen-Hee Kim, Dongsul Kim, Hyang Sook Chun

**Affiliations:** 1 Advanced Food Safety Research Group, BK21 Plus, School of Food Science and Technology, Chung-Ang University, Anseong 17546, Korea; ohe0904@hanmail.net (H.E.O.); TianFei@cau.ac.kr (F.T.); eyhongs@gmail.com (E.Y.H.); 2 Food Contaminants Division, National Institute of Food & Drug Safety Evaluation, Osong 28159, Korea; ojpaek92@korea.kr (O.P.); cinee@korea.kr (S.-H.K.); dongsul@korea.kr (D.K.)

**Keywords:** aflatoxins, sterigmatocystin, simultaneous determination, liquid chromatography–tandem mass spectrometry

## Abstract

An interlaboratory study was performed in eight laboratories to validate a liquid chromatography–tandem mass spectrometry (LC/MS/MS) method for the simultaneous determination of aflatoxins and sterigmatocystin (STC) in white rice and sorghum (*Sorghum bicolor*). Fortified samples (at three different levels) of white rice and sorghum were extracted, purified through a solid-phase extraction (SPE) column, and then analyzed by LC/MS/MS. The apparent recoveries (ARs) ranged from 78.8% to 95.0% for aflatoxins and from 85.3% to 96.7% for STC. The relative standard deviations for repeatability (RSD_r_) and reproducibility (RSDR) of aflatoxins were in the ranges 7.9%–33.8% and 24.4%–81.0%, respectively. For STC, the RSD_r_ ranged from 7.1% to 40.2% and the RSDR ranged from 28.1% to 99.2%. The Horwitz ratio values for the aflatoxins and STC ranged from 0.4 to 1.2 in white rice and from 0.3 to 1.0 in sorghum, respectively. These results validated this method for the simultaneous determination of aflatoxins and STC by LC/MS/MS after SPE column cleanup. The percentages of satisfactory *Z*-score values (|*Z*| ≤ 2) were the following: for white rice, 100% for aflatoxins and STC; for sorghum, 100%, except in data from two laboratories for STC (0.3 μg/kg). This validated that the LC/MS/MS method was successfully applied for the determination of aflatoxins and STC in 20 white rice and 20 sorghum samples sourced from Korean markets.

## 1. Introduction

Concern about human exposure to aflatoxins has tended inevitably to focus on high-risk commodities such as corn, nuts, and dried fruit, where levels of aflatoxins can be both variable and relatively high [1,2]. Although it is known that cereals can be contaminated with aflatoxins, the research emphasis, e.g., for wheat, has tended towards the monitoring and control of *Fusarium* toxins, where toxins such as deoxynivalenol commonly and frequently occur at mg/kg levels [3]. Although rice is not immediately thought of as a high-risk commodity in terms of contamination levels of aflatoxins, there is substantial evidence indicating endemic low concentration (mg/kg) occurrence of aflatoxin B_1_ contamination in rice [4,5]. Because rice is a staple food worldwide, low-level contamination can be of concern because it can lead to long-term exposure at above recommended levels. Sorghum is another important cereal crop worldwide. It is used in food items such as cookies, cakes, porridge, unleavened bread, and beverages [6]. Sorghum contains significant amounts of tannins in the testa, low amounts of phenolic acids in the grain [7], and abundant polyphenols, which enhance its resistance to pests and microbial infestation [8]. However, sorghum is vulnerable to fungal contamination. It has been estimated that economic losses in Asia and Africa due to fungal infestation were >$130 million annually [9]. *Aspergillus*, *Fusarium*, *Penicillium*, and *Alternaria* species are commonly detected in contaminated sorghum, and mycotoxigenic strains of these fungal species have been isolated from different sorghum varieties [9].

Aflatoxins is a family of structurally related mycotoxins that includes aflatoxin B_1_ (AFB_1_), aflatoxin B_2_ (AFB_2_), aflatoxin G_1_ (AFG_1_), and aflatoxin G_2_ (AFG_2_) [10]. These are the most important mycotoxins detected in food [11] and have been classified in group 1 as human carcinogens by the International Agency for Research on Cancer [12]. Many strains of *Aspergillus*, such as *Aspergillus flavus*, *Aspergillus parasiticus*, *Aspergillus bombycis*, *Aspergillus ochraceoroseus*, *Aspergillus nomius*, and *Aspergillus pseudotamari* can produce aflatoxins [13]. Maximum levels of aflatoxin contamination have been established by different countries to protect public health. In particular, the European Commission regulated 2 μg/kg for AFB_1_ and 4 μg/kg for total aflatoxins in cereals and derived products [14], and the Korea Ministry of Food and Drug Safety imposed limits of AFB_1_ < 10 μg/kg and total aflatoxins < 15 μg/kg [15].

Sterigmatocystin (STC) is a mycotoxin produced by the fungi of many different *Aspergillus* species. Other species such as *Bipolaris*, *Chaetomium*, and *Emericella* are also able to produce STC. STC-producing fungi have frequently been isolated from different foodstuffs. STC has been detected regularly in grains, corn, bread, cheese, spices, coffee beans, soybeans, pistachio nuts, animal feed, and silage [16]. STC exhibits various toxicological, mutagenic, and carcinogenic effects in animals, and it has been recognized as a 2B carcinogen (possible human carcinogen) by the International Agency for Research on Cancer [17]. Recent reviews described the occurrence of STC in a variety of foodstuffs [18,19,20]. The European Food Safety Authority (EFSA) Panel on Contaminants in the Food Chain (CONTAM Panel) analyzed a total of 1259 samples of cereal grains, cereal products, and nuts, collected between August 2013 and November 2014 in nine European countries (and originating from 45 countries), for the presence of STC. In cereal grains, STC was detected in 2%–6% of the wheat, rye, maize, and barley samples, mostly at levels < 1.5 μg/kg. A higher incidence and higher levels of contamination (14 samples, 1.5–6 µg/kg; 1 sample, 33 µg/kg) were observed in rice (in virtually all unprocessed rice and 21% of processed rice from the EU) and oats (22%) [21]. Sorghum is also commonly detected with STC contamination. Queslati et al. [22] analyzed 60 sorghum samples from Tunisian markets; 33% of them were detected with STC contamination at the mean level of 20.5 μg/kg. Chala et al. [23] detected similar levels of contamination (21.2 μg/kg) in 34% of the sorghum samples (70 samples) collected from Ethiopian markets; the highest concentration was 323 μg/kg.

Analytical methods involving chromatography have been developed for STC and aflatoxins. Thin-layer chromatography [24], high-performance liquid chromatography (HPLC) [25,26], and gas chromatography [27] have been used. More recently, liquid chromatography–mass spectrometry (LC/MS) methods were reported [28].

Liquid chromatography–tandem mass spectrometry (LC/MS/MS) has been shown to be suitable for the analysis of mycotoxins in cereals [29,30,31]; it enables simultaneous qualification and quantification. According to the EFSA [32], among the analytical methods available for the determination of STC, LC/MS methods demonstrate the limit of detection (LOD). However, the sensitivity of these methods depends on the matrices and methods used.

In the present study, we validated the method for quantifying aflatoxins and STC in white rice and sorghum (*Sorghum bicolor*) through an interlaboratory study. The method uses a single solid phase extract column for cleanup, and simultaneous determination by LC/MS/MS for the toxins.

## 2. Results

### 2.1. Collaborative Validation of LC/MS/MS Determination of Aflatoxins and STC in White Rice

The results of the interlaboratory study of aflatoxins and STC in white rice are tabulated in Table 1. The mean apparent recoveries (ARs) at all spike levels (0.3–10 μg/kg) were within the acceptable limits (70%–110%). However, the mean AR of aflatoxins (89%) was below the ARs of STC (94%) and the ARs of AFG_1_ and AFG_2_ (82%–88%) were noticeably lower in comparison with AFB_1_ and AFB_2_ (91%–93%). No outliers of aflatoxins or STC were observed for white rice. The relative standard deviations for repeatability (RSDr) and reproducibility (RSDR) for aflatoxins were 5.7%–18.6% and 26.0%–44.1%, respectively. A Horwitz ratio (HorRat) value in the range 0.5–2.0 was confirmed at all the spiked levels. Furthermore, values for RSDr, RSDR, and HorRat for STC were 5.2%–18.9%, 15.3%–21.9%, and 0.4 (<0.5), respectively.

### 2.2. Collaborative Validation Results of LC/MS/MS Determination of Aflatoxins and STC in Sorghum

The results of the interlaboratory study of aflatoxins and STC in sorghum are tabulated in Table 2. The mean ARs for aflatoxins and STC were in the ranges 79%–99% and 85%–97%, respectively. One outlier was observed in the sorghum sample spiked with 1.0 μg/kg of aflatoxins. The RSDr and RSDR for aflatoxins were in the ranges 8.1%–24.4% and 13.6%–42.5%, respectively. The HorRat values of aflatoxins ranged from 0.3 to 1.0. For STC, the AR, RSDr, and RSDR were in the ranges 85%–97%, 14.2%–29.0%, and 32.0%–54.7%, respectively. The HorRat values of STC ranged from 0.8 to 1.0.

### 2.3. Calculation of the Z-Scores

Eight Korean laboratories participated in the collaborative validation of aflatoxins and STC in white rice and sorghum. Calculation of the *Z*-scores to evaluate each laboratory’s proficiency was based on the results provided by the participating laboratories (Table 3 and Table 4). Eight laboratories completed the interlaboratory comparison for aflatoxins and STC successfully. Mean *Z*-scores were 0.58 and 0.49 for white rice and sorghum, respectively. All *Z*-scores for aflatoxins in both white rice and STC, which varied from –1.91 to 1.33, were within the acceptable limits (|*Z*| ≤ 2). The *Z*-scores reported for STC were within the range –2.19 to 2.82, with two unsatisfactory data (–2.19 and 2.82) at the 0.3 μg/kg level.

### 2.4. Application of the Validated LC/MS/MS Method to Market Samples

The validated LC/MS/MS method was used to determine aflatoxins and STC in 20 white rice and 20 sorghum samples sourced from Korean markets. Results are tabulated in Table 5. No rice samples were found to be contaminated with AFB_1_, AFB_2_, AFG_1_ or AFG_2_.

In sorghum samples, AFB_1_ was detected in 10% (2/20) of samples with values above the LOD. In one sorghum sample, AFB_1_ was detected between the LOD and limit of quantification (LOQ), and one sample was above the LOQ (detection range 0.1–1.0 μg/kg). AFG_2_ was detected in 15% (3/20) of sorghum samples with values above the LOD. In three samples, it was detected between the LOD and LOQ. No sorghum samples were detected with AFB_2_ or AFG_1_.

Regarding STC, it was detected in 70% (14/20) of white rice samples with values below the LOQ, while in 30% (6/20) it was detected between the LOD and LOQ. In contrast, STC was detected in only 10% (2/20) of sorghum samples. In two sorghum samples, STC and AFB_1_ were detected, but their concentrations were low (0.1–1.0 μg/kg). Furthermore, in one sorghum sample, STC, AFB_1_, and AFG_2_ were detected simultaneously.

## 3. Discussion

This study describes a harmonized collaborative validation of multiple mycotoxin detection in white rice and sorghum using LC/MS/MS. We found that the LC/MS/MS method possessed the performance characteristics required to obtain accurate results. In the case of white rice spiked with 1.0–10.0 μg/kg aflatoxins, the mean AR (82%–91%), and the values obtained for RSDr (5.7%–18.6%) and RSDR (26.5%–44.1%) were considered acceptable, taking into consideration the performance criteria (AR 70%–110% and RSD_R_ ≤ 45.2%) suggested by EU guidelines for aflatoxins [33]. The RSDR value (44.1%) of AFG_2_ spiked samples (5.0 µg/kg) was close to the maximum value stated by the EU guidelines. It might be caused by the sample-to-sample variation of matrix effects or the inter-laboratory variability result from different technical expertise and established workflows for individual laboratories. The important expression of the method’s precision is the HorRat value, which is calculated as a ratio of %RSDR to the predicted reproducibility RSD, %PRSDR. The %PRSDR is a function of the analyte concentration and is expressed as 2C^−0.1505^, where C is the estimated mean concentration [24]. In comparison with AOAC guidelines [24], a HorRat value in the range 0.5–2.0 was confirmed for white rice at all the spiked levels. This indicated that the presented method was reproducible for the determination of aflatoxins contained in white rice. HorRat values of STC were 0.4 (<0.5). Consistent deviations from the ratio that were on the low side (values < 0.5) may indicate unreported averaging or excellent training and experience [34].

For spiked sorghum, all parameters of aflatoxins satisfied the criteria mentioned [33] above, although one outlier (0.62 μg/kg) was observed in the AFB_1_ result at the 1.0 μg/kg level. Results also showed a HorRat value of 0.3 at the 1.0 μg/kg level of AFB_1_ in sorghum. However, no problems were recognized throughout this interlaboratory study, suggesting that outliers may have been due to random errors. It was also noticeable that the ARs of aflatoxins and STC in sorghum were slightly lower than they were in white rice, perhaps due to the matrix suppression effect of sorghum.

Calculation of the *Z*-scores suggested that all eight laboratories completed the interlaboratory comparison successfully. Unsatisfactory *Z*-scores for STC from individual laboratories were mainly a consequence of low or high ARs.

In summarizing information on analytical methods for STC in foodstuffs, Veršilovskis and de Saeger [19] concluded that LC/MS/MS should be used in efforts to develop sensitive methods and that sample preparation should be improved. This was realized in some new methods developed for the determination of STC in various grains and cheese by Veršilovskis et al. (LOD 0.03–0.15 μg/kg) [20,35] and in the multi-mycotoxin method reported by Sulyok et al. (LOD 0.4 μg/kg) [29]. Furthermore, Goto and co-workers [36] developed a new method for analyzing STC in grains using an immunoaffinity column (IAC) and LC/MS. This method is effective for STC analysis in grains and holds potential for a new application of a commercial IAC in STC analysis of aflatoxins. Based on EU guidelines, the AR, repeatability, and reproducibility levels were acceptable. Hence, this method should be useful for future studies on the occurrence and risk attached to aflatoxins and STC.

In our study, the validated LC/MS/MS method was successfully applied for the analysis of natural samples sourced from Korean markets. The results showed a low occurrence of aflatoxins in both white rice and sorghum samples, with no sample exceeding the EU maximum limits for aflatoxins (<2 μg/kg) [17,18]. AFB_1_ and AFG_2_ with concentrations > LOD values were only detected in sorghum samples. In contrast, the incidence of STC with concentrations < LOD values was high in sorghum samples, but their concentrations were low (0.1–1.0 μg/kg). Of particular importance was the simultaneous detection of STC, AFB_1_, and AFG_2_ in a sorghum sample.

## 4. Conclusions

Harmonized collaborative validation of aflatoxins and STC in white rice and sorghum was carried out using LC/MS/MS. The HorRat value was <1.5, which indicates that the method described in this study is reproducible for the determination of aflatoxins and STC contained in both white rice and sorghum at concentrations in the range 0.3–10.0 μg/kg. Currently, no country has legislation for STC. As aflatoxins and STC are naturally occurring substances, it is important to gather further information regarding suitable analytical methods for their detection. Our results indicate that the validated LC/MS/MS method described here can be successfully applied for the determination of aflatoxins and STC that naturally contaminate white rice and sorghum.

## 5. Materials and Methods

### 5.1. Reagents and Chemicals

Formic acid (LC/MS grade) was purchased from Fisher Scientific (Geel, Belgium). Water, acetonitrile, and methanol (HPLC grade) were purchased from J.T. Baker (Phillipsburg, NJ, USA). Mycotoxin solid standards AFB_2_ (5 mg, Lot No. A9887), AFG_1_ (1 mg, Lot No. A0138), and AFG_2_ (5 mg, Lot No. A0263) were purchased from Sigma-Aldrich (St. Louis, MO, USA). AFB_1_ (2.01 μg/mL in acetonitrile, Lot No. L15153A) and STC (50.2 μg/mL in acetonitrile, Lot No. L14424S) were purchased from Romer Labs (Tulln, Austria). For each aflatoxin and STC standard, a stock solution of 100 ng/mL was prepared in acetonitrile and stored at –20 °C. The aflatoxin solutions were calibrated spectrophotometrically at 350 nm, using the method published by the association of analytical communities (AOAC) [37]. Aflatoxins and STC working standard solutions were prepared by evaporating an exact volume of stock solution under nitrogen gas and re-dissolving the residue in acetonitrile to reach a final concentration of 10 ng/mL for AFB_1_, AFB_2_ and AFG_1_, 20 ng/mL for AFG_2_, and 3 ng/mL for STC, respectively.

### 5.2. Interlaboratory Study Design

Eight laboratories in Korea participated in this interlaboratory study to validate the method. The same aflatoxin and STC free samples of white rice and sorghum that were previously examined by a laboratory were spiked with aflatoxin and STC standard solutions to reach a final concentration of 1, 2.5 and 5 μg/kg for AFB_1_, AFB_2_, AFG_1_ and AFG_2_, and 0.3, 0.75 and 1.5 μg/kg for STC, respectively. All spiked samples were prepared by the organizer’s laboratory and sent to each participating laboratory for analysis. Each participant was supplied with three bottles of blank white rice and sorghum samples, and three bottles of spiked (3 levels) white rice and sorghum samples.

### 5.3. Sample Extraction

A spiked 5 g sample was placed in a 50 mL conical tube with 20 mL of extract solution (acetonitrile/water, 50:50, *v*/*v* containing 0.1% formic acid) and placed on a vortex shaker for 5 min. After extraction, the sample was centrifuged at 3500 rpm for 5 min and then filtered through glass microfiber filter paper (GF/A grade, 110 mm; Whatman, Little Chalfont, UK). A 4 mL volume of filtrate extract was diluted with 16 mL water. An SPE column (ISOLUTE^®^ Myco, 60 mg/3 mL; Biotage, Cardiff, UK) was preconditioned with 2 mL acetonitrile and then 2 mL water. Five milliliters of dilute solution were applied to the SPE column, followed by washing with 2 mL water and then 2 mL acetonitrile/water (10:90, *v*/*v*). The toxins that remained on the column were eluted with 4 mL acetonitrile. The collected eluate was evaporated with N_2_ gas under vacuum at 50 °C. The residue was dissolved in 1 mL acetonitrile/water (50:50, *v*/*v*) and then filtered through a polyvinylidene fluoride syringe filter (0.2 µm; Whatman, Pittsburgh, PA, USA).

### 5.4. Calibration Solutions

Aflatoxins and STC free samples of white rice and sorghum were used for the manufacture of matrix-matched calibration curves. Sample solutions were prepared using the same method described in sample extraction. Standard solution of mycotoxin was added into sample solution to reach different concentrations (0.5, 1.0, 2.5, 5.0, 10.0 μg/kg for AFB_1_, AFB_2_, and AFG_1_; 1.0, 2.0, 5.0, 10.0, 20.0 μg/kg for AFG_2_; and 0.15, 0.30, 0.75, 1.50, 3.00 μg/kg for STC, respectively). Calibration solutions were prepared by the organizer’s laboratory and sent to each participating laboratory for analysis. The concentrations of aflatoxins and STC in the sample solutions were calculated from calibration curves. The LOD and LOQ were calculated using the slope (*b*) of the matrix calibration curve and the residual standard error (*s_y/x_*) with the following equations [38]: LOD = 3.3 *s_y/x_/b*; LOQ = 10 *s_y/x_/b*. The LOQ for AFB_1_, AFB_2_, AFG_1_, AFG_2_, and STC was 0.28, 0.35, 0.42, 0.90, and 0.52 μg/kg, respectively.

### 5.5. LC/MS/MS Analysis

Each laboratory determined the aflatoxins and STC concentrations by LC/MS/MS. Two microliters of the final solution were loaded on an XBbridge C18 column (1.7 μm, 2.1 × 100 mm; Waters, Milford, MA, USA) at 35 °C. Gradient elution was carried out with mixtures of acetonitrile and water (see Table 6). Electrospray ionization in the positive mode was used. Multiple reactions monitoring (MRM) mode was used here with LC/MS/MS. All other experimental conditions were set by the respective participating laboratories. LC/MS/MS instruments and parameters used in the eight participating laboratories are tabulated in Table 7. Supplementary material, describing chromatograms of each mycotoxin detected by LC/MS/MS in MRM mode, is available (Figure S1).

### 5.6. Statistical Analysis

The results received from the participating laboratories were initially evaluated for evidence of outliers using the following statistical tests: the Cochran test and the single value and pair value Grubbs tests (between laboratory means) [39]. The RSD_r_ and RSDR, and the HorRat value (the ratio of the RSDR to the predicted RSDR) were obtained using an analysis of variance according to AOAC guidelines [24]. The criteria for analytical methods mentioned in Commission Regulation (EC) No. 401/2006 were used for evaluation of these parameters [33]. The *Z*-score compares the analytical result to the assigned value and can be used to describe the comparability of results. The *Z*-score was derived from the results of each participant according to the following equation [40]:

$$Z-\mathrm{score}\;=\frac{X_{\mathrm{lab}}–X_{\mathrm{ref}}}{\sigma_{p}},$$

where X_lab_ is the result reported by the participant, expressed as a dimensionless mass ratio, e.g., 1 μg/kg = 10^−9^; X_ref_ is the assigned value expressed as a dimensionless ratio, e.g., 1 μg/kg = 10^−9^; and σ_P_ is the target standard deviation (SD) for proficiency assessment. The target SD (σ_p_) was calculated from the modified Horwitz equation:

$$\sigma_{p}=\frac{0.22\times c}{mr},$$

where c is the concentration of the assigned value, expressed as a dimensionless mass ratio, e.g., 1 μg/kg = 10^−9^; and mris the dimensionless mass ratio, 1 μg/kg = 10^−9^. The *Z*-score classification was as follows: |*Z*| ≤ 2, acceptable; 2 < |*Z*| ≤ 3, questionable; |*Z*| > 3, unacceptable.

## Figures and Tables

**Table 1:** Collaborative validation results of liquid chromatography–tandem mass spectrometry (LC/MS/MS) determination of aflatoxins and sterigmatocystin (STC) in white rice.

Laboratory	Spiked Samples (μg/kg)														
	AFB_1_ *^a^*			AFB_2_ *^b^*			AFG_1_ *^c^*			AFG_2_ *^d^*			STC *^e^*		
	1.0	2.5	5.0	1.0	2.5	5.0	1.0	2.5	5.0	2.0	5.0	10.0	0.30	0.75	1.50
1	1.09	2.54	5.02	1.08	2.55	5.13	1.07	2.54	5.03	2.00	5.06	10.78	0.30	0.71	1.55
2	0.69	1.51	3.24	0.87	1.67	3.58	0.62	1.56	3.13	1.41	3.01	6.48	0.26	0.61	1.19
3	0.76	1.96	3.96	0.76	1.89	3.85	0.63	1.80	3.71	1.38	3.23	7.04	0.29	0.69	1.37
4	0.95	2.56	4.68	0.98	2.57	4.99	0.91	2.14	3.86	1.73	3.05	7.65	0.22	0.65	1.24
5	0.99	2.43	4.76	1.04	2.54	4.90	0.95	2.48	4.98	2.10	5.26	9.93	0.28	0.68	1.35
6	1.01	2.63	5.02	0.95	2.58	5.14	1.04	2.72	5.40	2.03	5.17	10.37	0.29	0.70	1.38
7	0.69	1.78	3.91	0.49	1.53	3.74	0.60	1.58	3.77	1.28	2.72	6.94	0.34	0.75	1.55
8	1.13	2.84	5.65	1.11	2.79	5.70	1.08	2.71	5.27	2.03	5.26	11.10	0.33	0.80	1.53
Mean (μg/kg)	0.91	2.28	4.53	0.91	2.27	4.63	0.86	2.19	4.39	1.75	4.10	8.79	0.29	0.70	1.39
AR (%) *^f^*	91.0	91.2	90.6	91.0	90.8	92.6	86.0	87.6	87.8	87.5	82.0	87.9	96.7	93.3	92.7
Outlier *^g^*	0	0	0	0	0	0	0	0	0	0	0	0	0	0	0
RSD_r_ (%) *^h^*	16.4	5.7	9.3	16.9	13.4	7.3	18.1	15.6	9.4	18.6	13.3	10.9	15.9	18.9	5.2
RSDR (%) *^i^*	31.2	31.2	26.5	35.9	33.7	26.0	39.1	35.4	30.3	32.1	44.1	34.0	21.9	18.4	15.3
HorRat	0.7	0.8	0.7	0.8	0.9	0.7	0.9	0.9	0.9	0.8	1.2	1.1	0.4	0.4	0.4

^*a*^ AFB_1_: aflatoxin B_1_; ^*b*^ AFB_2_: aflatoxin B_2_; ^*c*^ AFG_1_: aflatoxin G_1_; ^*d*^ AFG_2_: aflatoxin G_2_; ^*e*^ STC: sterigmatocystin; ^*f*^ AR: apparent recovery; ^*g*^ Outlier: cochran and single Grubbs parameters; ^*h*^ RSD_r_: relative standard deviation of repeatability; ^*i*^ RSDR: relative standard deviation of reproducibility.

**Table 2:** Collaborative validation results of LC/MS/MS determination of aflatoxins and STC in sorghum.

Laboratory	Spiked Samples (μg/kg)														
	AFB_1_ *^a^*			AFB_2_ *^b^*			AFG_1_ *^c^*			AFG_2_ *^d^*			STC *^e^*		
	1.0	2.5	5.0	1.0	2.5	5.0	1.0	2.5	5.0	2.0	5.0	10.0	0.30	0.75	1.50
1	1.00	2.44	4.64	0.89	2.04	4.79	0.95	2.24	4.77	1.81	4.26	9.50	0.21	0.51	1.06
2	1.03	1.69	2.87	0.74	1.55	2.94	0.79	1.71	2.98	2.22	3.50	6.03	0.14	0.35	0.76
3	1.10	2.39	4.56	0.93	2.22	4.23	1.01	2.25	4.31	0.82	3.38	7.67	0.27	0.65	1.45
4	0.62	1.84	3.49	0.71	1.86	4.46	0.58	2.46	4.24	1.28	3.96	9.18	0.25	0.66	1.35
5	0.90	2.35	4.88	0.93	2.19	4.88	0.89	2.23	4.68	1.87	4.31	9.36	0.30	0.72	1.46
6	1.04	2.62	5.40	1.07	2.57	5.42	1.01	2.55	5.68	2.01	4.75	10.34	0.32	0.76	1.56
7	0.97	1.94	3.96	0.93	1.83	4.04	0.50	1.43	3.63	1.54	3.04	7.10	0.47	0.72	1.23
8	0.92	2.37	3.95	0.92	2.42	3.46	0.91	2.32	3.48	1.65	4.31	6.38	0.33	0.77	1.39
Mean (μg/kg)	0.99	2.21	4.22	0.89	2.08	4.28	0.83	2.15	4.22	1.65	3.94	8.19	0.29	0.64	1.28
AR (%) *^f^*	99.0	88.4	84.4	89.0	83.2	85.6	83.0	86.0	84.4	82.5	78.8	81.9	96.7	85.3	85.3
Outlier *^g^*	1	0	0	0	0	0	0	0	0	0	0	0	0	0	0
RSD_r_ (%) *^h^*	11.9	14.6	8.1	18.3	17.4	9.6	21.6	14.6	17.4	19.3	24.4	12.8	29.0	24.2	14.2
RSDR (%) *^i^*	13.6	24.8	29.4	23.0	26.9	28.8	38.5	28.6	32.7	42.5	28.1	30.8	54.7	38.0	32.0
HorRat	0.3	0.6	0.8	0.5	0.7	0.8	0.9	0.7	0.9	1.0	0.8	1.0	1.0	0.8	0.8

^*a*^ AFB_1_: aflatoxin B_1_; ^*b*^ AFB_2_: aflatoxin B_2_; ^*c*^ AFG_1_: aflatoxin G_1_; ^*d*^ AFG_2_: aflatoxin G_2_; ^*e*^ STC: sterigmatocystin; ^*f*^ AR: apparent recovery; ^*g*^ Outlier: cochran and single Grubbs parameters; ^*h*^ RSD_r_: relative standard deviation of repeatability; ^*i*^ RSDR: relative standard deviation of reproducibility.

**Table 3:** Results of mycotoxin analysis and relevant scoring in white rice.

Toxins	Assigned Values (μg/kg)	Laboratory							
		1	2	3	4	5	6	7	8
AFB_1_ *^a^*	1	0.79	−1.03	−0.70	0.16	0.34	0.43	−1.00	0.99
	2.5	0.47	−1.40	−0.57	0.50	0.27	0.63	−0.91	1.02
	5	0.45	−1.17	−0.52	0.13	0.21	0.45	−0.56	1.02
AFB_2_ *^b^*	1	0.76	−0.18	−0.70	0.32	0.61	0.19	−1.91	0.92
	2.5	0.52	−1.08	−0.69	0.56	0.50	0.58	−1.34	0.96
	5	0.46	−0.95	−0.71	0.33	0.25	0.46	−0.80	0.97
AFG_1_ *^c^*	1	0.95	−1.12	−1.05	0.23	0.40	0.82	−1.20	0.97
	2.5	0.64	−1.14	−0.71	−0.09	0.52	0.96	−1.12	0.95
	5	0.58	−1.15	−0.62	−0.48	0.54	0.91	−0.57	0.79
AFG_2_ *^d^*	2	0.58	−0.77	−0.82	−0.04	0.80	0.64	−1.05	0.65
	5	0.87	−0.98	−0.78	−0.95	1.06	0.98	−1.25	1.06
	10	0.91	−1.05	−0.80	−0.51	0.52	0.72	−0.84	1.05
STC *^e^*	0.3	0.17	−0.40	−0.05	−0.98	−0.09	−0.01	0.79	0.57
	0.75	0.04	−0.54	−0.03	−0.32	−0.10	0.02	0.31	0.63
	1.5	0.48	−0.61	−0.08	−0.46	−0.15	−0.05	0.47	0.40

*^a^* AFB_1_: aflatoxin B_1_; *^b^* AFB_2_: aflatoxin B_2_; *^c^* AFG_1_: aflatoxin G_1_; *^d^* AFG_2_: aflatoxin G_2_; *^e^* STC: sterigmatocystin.

**Table 4:** Results of mycotoxin analysis and relevant scoring in sorghum.

Toxins	Assigned Values (μg/kg)	Laboratory							
		1	2	3	4	5	6	7	8
AFB_1_ *^a^*	1	0.26	0.38	0.69	−1.50	−0.23	0.41	0.11	−0.12
	2.5	0.43	−0.93	0.34	−0.66	0.26	0.75	−0.48	0.30
	5	0.38	−1.22	0.31	−0.66	0.60	1.07	−0.24	−0.25
AFB_2_ *^b^*	1	0.02	−0.68	0.17	−0.80	0.17	0.80	0.16	0.15
	2.5	−0.09	−0.97	0.24	−0.41	0.20	0.88	−0.46	0.60
	5	0.47	−1.22	−0.04	0.17	0.54	1.04	−0.21	−0.74
AFG_1_ *^c^*	1	0.54	−0.19	0.84	−1.14	0.27	0.82	−1.52	0.38
	2.5	0.17	−0.79	0.18	0.57	0.15	0.73	−1.31	0.31
	5	0.50	−1.13	0.08	0.02	0.42	1.33	−0.54	−0.67
AFG_2_ *^d^*	2	0.36	1.31	−1.89	−0.84	0.50	0.82	−0.25	0.00
	5	0.29	−0.40	−0.51	0.01	0.34	0.74	−0.82	0.34
	10	0.59	−0.98	−0.24	0.45	0.53	0.98	−0.50	−0.82
STC *^e^*	0.3 *^f^*	−1.17	−2.19	−0.32	−0.49	0.25	0.51	2.82	0.60
	0.75	−0.81	−1.79	0.06	0.12	0.47	0.71	0.44	0.79
	1.5	−0.67	−1.57	0.50	0.19	0.55	0.84	−0.17	0.33

*^a^* AFB_1_: aflatoxin B_1_; *^b^* AFB_2_: aflatoxin B_2_; *^c^* AFG_1_: aflatoxin G_1_; *^d^* AFG_2_: aflatoxin G_2_; *^e^* STC: sterigmatocystin; *^f^* Two laboratories had 2 < |*Z*| ≤ 3.

**Table 5:** Application of validated LC/MS/MS for determination of aflatoxins and sterigmatocystin in rice and sorghum samples sourced from Korean markets.

Myotoxin	Product	Sample	Number of Samples			Concentration Range *^h^* (μg/kg)	
			<LOD *^f^*	LOD–LOQ *^g^*	>LOQ	0.1~1.0	1.0~2.0
AFB_1_ *^a^*	Rice	20	20 (100) *^i^*	-	-	-	-
	Sorghum	20	18 (90)	1 (5)	1 (5)	2 (10)	-
AFB_2_ *^b^*	Rice	20	20 (100)	-	-	-	-
	Sorghum	20	20 (100)	-	-	-	-
AFG_1_ *^c^*	Rice	20	20 (100)	-	-	-	-
	Sorghum	20	20 (100)	-	-	-	-
AFG_2_ *^d^*	Rice	20	20 (100)	-	-	-	-
	Sorghum	20	17 (85)	3 (15)	-	-	-
STC *^e^*	Rice	20	14 (70)	6 (30)	-	-	-
	Sorghum	20	-	18 (90)	2 (10)	2 (10)	-

*^a^* AFB_1_: aflatoxin B_1_; *^b^* AFB_2_: aflatoxin B_2_; *^c^* AFG_1_: aflatoxin G_1_; *^d^* AFG_2_: aflatoxin G_2_; *^e^* STC: sterigmatocystin; *^f^* LOD: limit of detection; *^g^* LOQ: limit of quantification; *^h^* AF concentration was not corrected for the AR result; *^i^* Figure in parenthesis indicates percentage.

**Table 6:** Analysis of aflatoxins and sterigmatocystin residues by LC/MS/MS.

Time, min *^a^*	A, % *^b^*	B, % *^c^*
0.0	90	10
3.0	90	10
10.0	30	70
10.1	10	90
12.0	10	90
12.1	90	10
15.0	90	10

*^a^* Flow rate, 0.2 mL/min; *^b^* Mobile phase A, 0.1% formic acid in water; *^c^* Mobile phase B, 0.1% formic acid in acetonitrile.

**Table 7:** LC/MS/MS instruments and parameters used in the eight participating laboratories.

Lab.	LC-MS/MS Equipment	AFB_1_ *^a^*		AFB_2_ *^b^*		AFG_1_ *^c^*		AFG_2_^d^		STC *^e^*	
		Precursor Ion (*m*/*z*)	Product Ion (*m*/*z*) *^f^*	Precursor Ion (*m*/*z*)	Product Ion (*m*/*z*)	Precursor Ion (*m*/*z*)	Product Ion (*m*/*z*)	Precursor Ion (*m*/*z*)	Product Ion (*m*/*z*)	Precursor Ion (*m*/*z*)	Product Ion (*m*/*z*)
1	ThermoAccela TSQ Quantum Ultra (Thermo Scientific, Waltham, MA, USA)	313.0 [M + H]^+^	284.9	315.0 [M + H]^+^	287.0	329.1 [M + H]^+^	311.0	331.1 [M + H]^+^	313.0	325.1 [M + H]^+^	310.0
2	HPLC coupled with TSQ Quantum (Thermo Scientific, Waltham, MA, USA)		241.1		259.0		199.8		245.0		310.0
3	ThermoAccela TSQ Quantum Ultra (Thermo Scientific, Waltham, MA, USA)		284.9		287.0		311.0		313.0		310.0
4	Acquity I-class UPLC Xevo TQ-S (Waters, Milford, MA, USA)		241.1		259.0		199.8		245.0		310.0
5	Acquity I-class UPLC Xevo TQ-2 (Waters, Milford, MA, USA)		284.9		287.0		243.0		245.0		281.0
6	ThermoAccela TSQ Quantum Ultra (Thermo Scientific, Waltham, MA, USA)		284.9		287.0		311.0		313.0		310.0
7	Acquity I-class UPLC Xevo TQ-S (Waters, Milford, MA, USA)		241.1		259.0		199.8		245.0		310.0
8	Acquity I-class UPLC Xevo TQ-2 (Waters, Milford, MA, USA)		241.1		259.0		199.8		245.0		310.0

*^a^* AFB_1_: aflatoxin B_1_; *^b^* AFB_2_: aflatoxin B_2_; *^c^* AFG_1_: aflatoxin G_1_; *^d^* AFG_2_: aflatoxin G_2_; *^e^* STC: sterigmatocystin; *^f^* In addition to the product ion selected in each laboratory for quantification.

## Supplementary Materials

The following are available online at www.mdpi.com/2072-6651/8/12/371/s1, Figure S1: Chromatograms of multimycotoxins: (A) aflatoxin B1; (B) aflatoxin B2; (C) aflatoxin G1; (D) aflatoxin G2; and (E) STC.
