# Supplementary material for: Harmonized Collaborative Validation of Aflatoxins and Sterigmatocystin in White Rice and Sorghum by Liquid Chromatography Coupled to Tandem Mass Spectrometry
Source: Toxins (Basel). 2016 Dec 13;8(12):371. doi: 10.3390/toxins8120371 (PMC5198565; doi:10.3390/toxins8120371)
Supplement: Supplementary file 1 [file toxins-08-00371-s001.pdf]

Supplementary Materials: Harmonized Collaborative Validation of Aflatoxins and Sterigmatocystin in White Rice and Sorghum by Liquid Chromatography Coupled to Tandem Mass Spectrometry

Hyun Ee Ok, Fei Tian, Eun Young Hong, Ockjin Paek, Sheen-Hee Kim, Dongsul Kim and Hyang Sook Chun

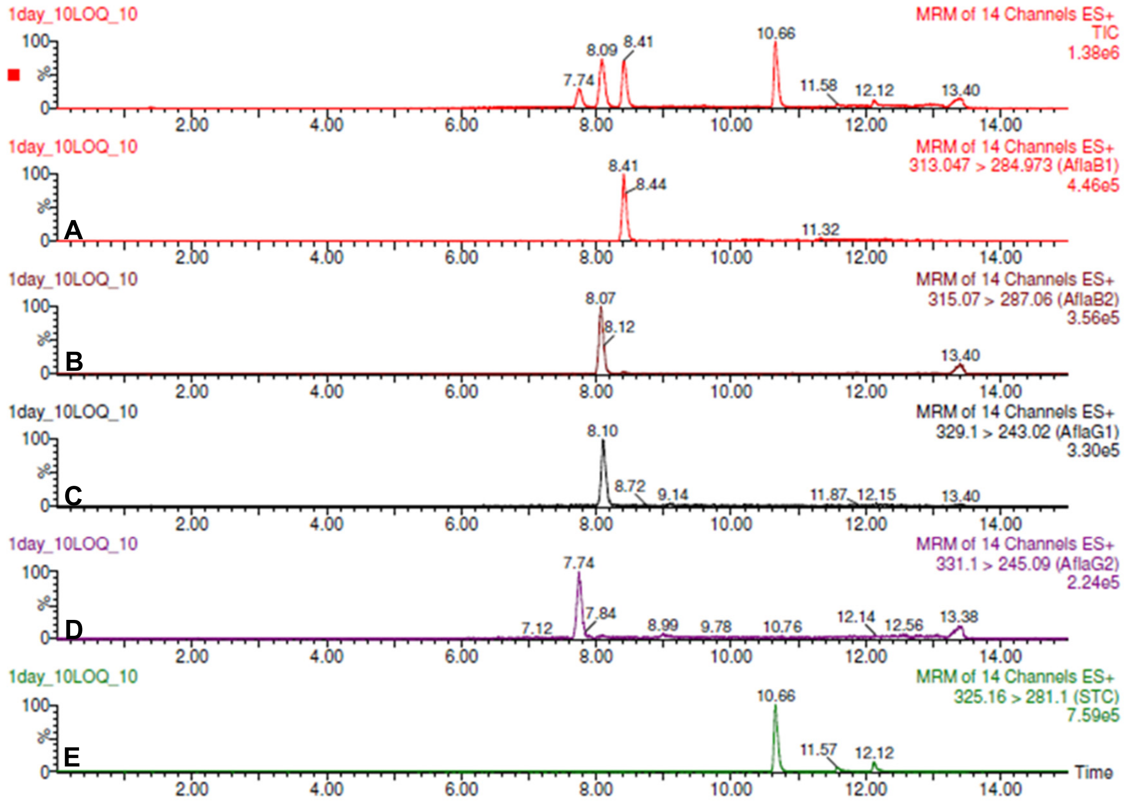

Figure S1. Chromatograms of multimycotoxins: (**A**) aflatoxin B_1; (**B**) aflatoxin B_2; (**C**) aflatoxin G_1; (**D**) aflatoxin G_2; and (**E**) STC.
